# Alterations in hippocampal neurogenesis and hippocampal insulin signaling pathway in rat with metabolic syndrome

**DOI:** 10.22038/IJBMS.2022.64917.14295

**Published:** 2022-11

**Authors:** Pinar Bayram, Deniz Billur, Sule Kizil, Hasan Caliskan, Belgin Can

**Affiliations:** 1 Kafkas University, Faculty of Medicine, Department of Histology and Embryology, Kars, Turkey; 2 Ankara University, Faculty of Medicine, Department of Histology and Embryology, Ankara, Turkey; 3 Lokman Hekim University, Faculty of Medicine, Department of Histology and Embryology, Ankara, Turkey; 4 Balıkesir University, Faculty of Medicine, Department of Physiology, Balıkesir, Turkey

**Keywords:** Behavior tests, Hippocampus, Immunohistochemistry, Insulin, Metabolic syndrome, Neurogenesis

## Abstract

**Objective(s):**

We aimed to examine the level of hippocampal neurogenesis, and assess learning and anxiety and the level of some proteins involving insulin signaling pathways in rats with Metabolic Syndrome (MetS); and to reveal the relationship among them.

**Materials and Methods:**

Totally, 30 Wistar-albino rats were used. The rats were divided into three groups: Control, MetS, and MetS+Ins. Immunohistochemical staining was performed to evaluate the levels of neurogenesis markers; Doublecortin (DCX), Neuronal-Differentiation-1 (NeuroD1), Ki67, and Neuronal nuclear protein (NeuN). Then, cleaved caspase-3 and TUNEL labeling were performed to detect the level of apoptosis. Additionally, behavior tests were performed to evaluate the learning-memory levels and anxiety-like behaviors. Insulin, Insulin Receptor (IR), Insulin Receptor Substrate (IRS2), glucose transporter (GLUT)-3, and GLUT4 protein expression levels were analyzed to evaluate the possible changes in the insulin signaling pathway.

**Results:**

An increase in anxiety with memory deficiency was observed in MetS. In the hippocampus of MetS, an increase was detected in the level of apoptosis, whereas a decrease was detected in the expression level of the neurogenesis marker. Insulin secretion and IR levels decreased in hippocampal neurons. We observed that GLUT3 and GLUT4 levels increased because of the non-activated insulin signaling pathway.

**Conclusion:**

We think that the insulin signaling pathway may have an effect on the decreased neurogenesis in the MetS group. So, the evaluation of the Mitogen-activated protein kinase (MAPK) pathway and the investigation of the effect of endoplasmic reticulum stress on this pathway will be among the targets of our future studies.

## Introduction

Metabolic syndrome (MetS) has been defined as a cluster of systemic disorders such as abdominal obesity, glucose intolerance or diabetes mellitus (DM), dyslipidemia, hypertension, and coronary artery disease added together with insulin resistance (1). On the other hand, insulin resistance is the main pathological feature of MetS, which consists of a number of disorders such as DM, obesity, dyslipidemia, and hypertension. Recent studies reveal that insulin resistance also develops in the nervous system. Also, it has been demonstrated that fetal neuron cultures produce and release insulin-like mRNA and insulin-like substances, which are not different from real insulin (2).

One of the places where adult neurogenesis occurs is the subgranular zone (SGZ), which is the lower border of the hippocampal dentate gyrus. Type1 cells in SGZ are neural stem cells and differentiate up to Type 5 cells (3). Most of the proliferative activity in adult SGZ occurs in Type 2 cells. Type 2b and Type 3 cells express Neuro D1 and DCX (4), while Type 4 and Type 5 cells express NeuN (5).

Hippocampus is one of the areas in the brain where insulin receptors are abundant. Insulin binds mostly to the molecular cell layer of the dentate gyrus and the pyramidal cells of the CA1 region in the hippocampus (6). Dysregulation of insulin receptor signaling in the brain has been shown to be associated with neurodegenerative diseases, dementia, and mood disorders (7). By binding insulin to the insulin receptor (IR), the receptor is autophosphorylated, followed by phosphorylation of insulin receptor substrate (IRS) proteins (8). IRS1 and IRS2 are widely expressed in the brain. With phosphorylation of IRS proteins, phosphatidyl-inositol-3 kinase (PI3K) pathways contribute to the normal functions and survival of neuronal cells (9).

In addition, it has been shown that recognition memory impairments occur with the deletion of IR and IGF1R in the hippocampus and amygdala, and spatial learning is impaired with the loss of IR in the hippocampus (10). Recent studies have shown that deletion of IRS2 in male mice reveals a negative effect on hippocampus-related emotional responses and spatial memory (11). 

GLUT 3 is the major glucose transporter in neurons of the hippocampus, cerebellum, striatum, and cortex (12) and is insulin-independent (13). Unlike peripheral tissues, GLUT4 is present at low levels in the brain and is not significantly regulated by insulin. GLUT4 is localized in the olfactory bulb, dentate gyrus (DG) of the hippocampus, hypothalamus, and cortex, but not as high as GLUT1 and GLUT3, is found in both the plasma membrane and the cytoplasm and is easily localized to the membrane when needed (2).

In this study, we aimed to examine the level of hippocampal neurogenesis, and assess learning and anxiety and the level of some proteins involving insulin signaling pathways in rats with MetS, and to reveal the relationship among them.

## Materials and Methods

***Experimental protocols***

This study was approved by Experimental Animals Local Ethics Committee, Ankara University, Türkiye (number 2017-16-133). Thirty, 12-week-old, 250–300 g, male Wistar albino rats were purchased and used in this study. The rats were randomly divided into three groups; Group I (Control, n=10); Group II (MetS, n=10), and Group III (MetS+Ins, n=10). All animals were exposed to 12 hr light/dark cycle. Food and water were available *ad libitum*.

Tap water was given to rats in the control group, while tap water containing 32% (935 mM) sucrose was given to rats in MetS and MetS+Ins groups for 20 weeks. Body weight measurements, fasting blood glucose levels, oral glucose tolerance test values, serum insulin levels (Elabscience Rat Insulin ELISA Kit, Cat no: E-EL-R2466), and tail arterial blood pressure measurements (NIBP200-A noninvasive blood pressure monitor, BIOPAC) were performed for the validation of MetS experimental model. After validation, 100 U/ml of subcutaneous insulin (Humalog Mix 25 Kwikpen 100 U/ml, Lilly İlaç Ticaret Ltd. Şti.) was injected into rats in the MetS+Ins group for two weeks (14).

***Animal behavior testing***

All behavioral experiments were carried out in Banu Ocakçıoğlu Learning and Memory Laboratory, Faculty of Medicine, Ankara University, Türkiye. The laboratory was silent and had a constant temperature (22 ± 2 °C). The tests were always conducted in the early morning (08:00 am –12:00 am) in decent light (110 lx, warm light). To evaluate learning and memory levels, Elevated Plus Maze Test (EPM), and Vanderwolf Swimming Test; and to assess anxiety-like behaviors Open Field Test (OFT), EPM, and Light Dark Box (LDB) Test were performed (15). 

For EPM, the experimental setup consisted of two open and two closed arms arranged in a plus (+) shape, with a height between 50 and 70 cm from the ground. If the animals spent more time in the closed arm, it was indicative of increased anxiety, if they spent more time in the open arm, it was indicative of decreased anxiety. This system is used to measure anxiety caused by fear of heights in rats and mice. 

On the other hand, Vanderwolf is a swimming test for evaluating spatial learning. Test duration was two days. In the experimental setup, there was a water tank and a platform placed in the water tank. The animals were trained 10 times on the first day and 10 times on the second day in the swimming tank. These workouts lasted for a maximum of one minute. The time for the animals to climb the platform on the first and second days was determined and this training was recorded with a camera.

The OFT is a test used to measure anxiety resulting from both the new environment and the fear of open fields. The system is square/cylindrical and contains 20 squares. As the anxiety increases, the animals spend their time in the squares usually located at the edges, and as the anxiety decreases the animals spend their time in the squares located in the middle parts of the system. The locomotor activity increases parallel with the decrease of anxiety in animals. The open field test was applied for five minutes.

The experimental setup of LDB consists of two equal chambers in contact with each other, measuring 110 x 40 cm. One of these compartments is the bright area and the other is the dark area, and there is a hole in the middle that connects the two areas. The animals were placed in the bright area and observed for five minutes. The behavior of the animals during the experiment was recorded with a camera. 

***Immunohistochemical staining***

The animals were deeply anesthetized using ketamine/xylazine (90 mg/kg–10 mg/kg, intramuscular). After decapitation, the brains were removed and fixed in 10% buffered formalin for immunohistochemistry markings and embedded in paraffin blocks. The sections were boiled with citrate buffer solution in the microwave for antigen retrieval and washed with phosphate-buffered saline (PBS). To prohibit endogenous peroxidase activity, the tissues were treated with 3% H_2_O_2_, then washed with PBS, and then incubated with a blocking solution (Thermo Scientific, TA-060-PBQ) for 5 min at room temperature. The sections were incubated overnight at 4°C with the following primary antibodies; DCX (sc-390645, 1/100 dilution), NeuroD1 (ab109224, 1/100 dilution), Ki67 (PA5-19462, 1/100 dilution, NeuN (ab177487, 1/1000 dilution), insulin (Thermo #14-9769-82, 1/200 dilution), IR (ab203746, 1/200 dilution), IRS2 (sc-390761HRP, 1/150 dilution), GLUT3 (sc-74399HRP, 1/200 dilution), GLUT4 (sc-53566, 1/200 dilution), and cleaved Caspase-3 (CST #9661, 1/150 dilution). After incubation with primary antibodies, the sections were washed with PBS and incubated with secondary antibodies (Thermo Scientific, TA-060-PB) for 10 min at room temperature. After the secondary antibody was washed with PBS, the sections were incubated with streptavidin peroxidase for 10 min at room temperature. 3,3-diaminobenzidine (DAB) solution was applied on the sections as chromogen for 2 min. Then, dH_2_O was used to stop the DAP reaction. The sections were dehydrated with alcohol series and covered with Entellan. Photomicrographs of six different areas were taken from each preparation at 40X magnification. For H-Score analysis (insulin, IR, IRS2, GLUT3, and GLUT4), ImageJ software (ImageJ1, 51j8, National Institutes of Health, USA) was used and the staining intensity was determined as negative: 0, low positive: 1, positive: 2, high positive: 3. Pixel ratios (Pi) were determined for the staining intensities between 0–100%. The obtained rates were digitized using the formula H-Score=ΣPi (i+1) (16). In the formula, “1” was used for correction of optical density. 

***TUNEL (Terminal deoxynucleotidyl transferase dUTP nick end labeling) staining***

The sections were incubated with TUNEL (Roche, Catalogue number: 11684817910) reaction mixture (labeling solution+terminal deoxynucleotidyl transferase enzyme solution) for 60 min at 37 °C and washed with PBS. For negative control, only label solution (without terminal transferase) was used instead of the TUNEL reaction mixture. After this step, the sections were incubated with Converter-POD for 30 min at 37 °C and then washed with PBS. The sections were incubated with DAB at room temperature for 2 min. Then, the sections were dehydrated with alcohol series and covered with Entellan. To determine the positive immunostaining cells, the area of the DG and/or SGZ was calculated. The number of positive cells (DCX, NeuroD1, Ki67, NeuN, cleaved caspase-3, and TUNEL) was calculated in the area of 1000 µm^2^.

***Statistical analysis***

IBM SPSS 22.0 program was used for the statistical evaluation of IHC analyzes. The difference among the groups was evaluated with One-Way ANOVA for those with normal distribution, and Tukey *post hoc* multiple comparisons test was applied for the difference between the pairs. In our study, the Kruskal Wallis test was used to evaluate the data which did not show normal distribution. The mean and standard deviation (mean ±SD) are given as descriptive statistics. The value of *P*<0.05 was considered statistically significant for the results.

## Results

***Validation of MetS experimental model in rats***

**Figure 1:**
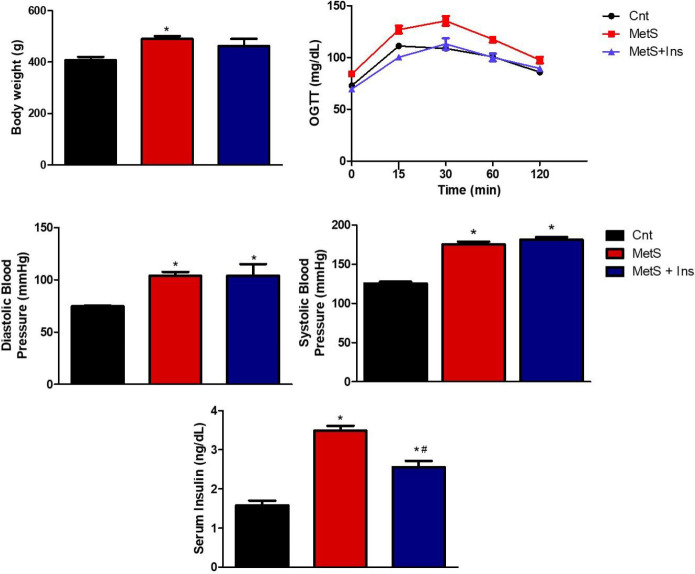
General parameters for validation of the MetS experimental model and after insulin administration. A) Changes in body weight in all groups. B) OGTT by measuring blood glucose levels before and after 1 g/kg glucose administration at 15^th^ min, 30^th^ min, 60^th^ min, and 120^th^ min to the rats. C) Diastolic blood pressure changes measured from the tail among the groups. D) Systolic blood pressure changes measured from the tail among the groups. E) Serum insulin levels in all groups (ng/ml)

Animals were fed with tap water containing 32% sucrose to create the MetS experimental model. Fasting blood glucose levels were regularly measured starting from the 12^th^ week. Significant results were obtained in the 20^th^ week (*P*=0.0001). After 20 weeks, rats in the Mets group had a weight gain of approximately 20%, when compared with the control group (Figure 1A). The development of insulin resistance in rats was detected using the oral glucose tolerance test (OGTT). Blood glucose levels were increased following administration of 1 g/kg glucose in both MetS and MetS+Ins groups (Figure 1B). Tail blood pressure was measured to evaluate changes in arterial blood pressure, which is among the necessary criteria for the validation of the MetS experimental model. An increase was observed in the MetS group in terms of diastolic (Figure 1C) and systolic (Figure 1D) blood pressure (40% and 36%, respectively) (*P*<0.0001). An approximately 120% (3.48 ng/ml) increase was detected in serum insulin levels of the MetS group (Figure 1E). The obtained data were parallel with current literature (14, 17). 

After insulin administration, approximately 8% weight loss was detected in the MetS+Ins group. Finally, fasting blood glucose level was significantly lower (*P*=0.0004), serum insulin levels decreased by approximately 32% (2.37 ng/ml), systolic blood pressure levels increased by 9.5% (*P*<0.0642), and diastolic blood pressure levels did not change in the MetS+Ins group.

***Neurogenesis was decreased in MetS***

**Figure 2:**
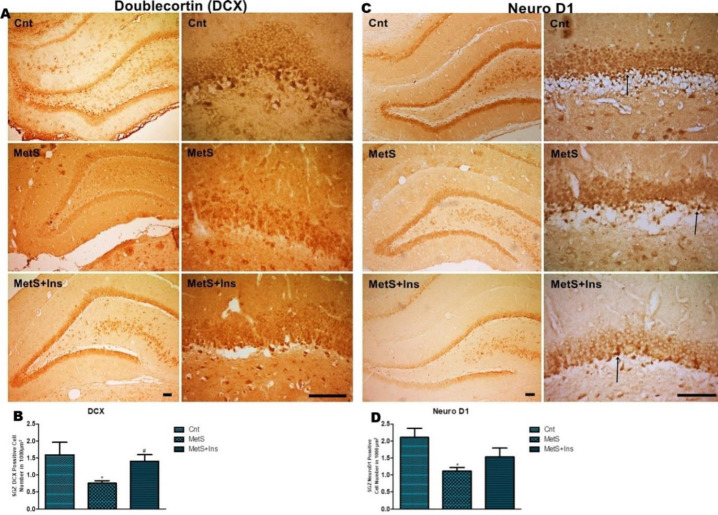
Immunohistochemical staining of the DCX and Neuro D1 positive cells in the hippocampus. A) DCX-positive cells in SGZ were stained dark brown and were shown with an arrow. B) DCX positive cell numbers in 1000 µm^2^ in SGZ were shown with a graph. C) NeuroD1 positive cells in SGZ were stained dark brown and were shown with an arrow. D) Positive cell number in 1000 µm^2^ in SGZ was shown in the graph

A significant difference was detected between the groups in terms of the number of DCX, NeuroD1, and Ki67 positive cells [F(2.19)=5.799 *P*=0.011, F(2.32)=4.956 *P*=0.013, F(2.30)=4.945 *P*=0.014, respectively], as seen in Figure 2A-C. There were no statistically significant differences between control and MetS+Ins groups (*P*=0.598) and between MetS and MetS+Ins groups (p=0.130) (Figure 2B and 2D).

**Figure 3:**
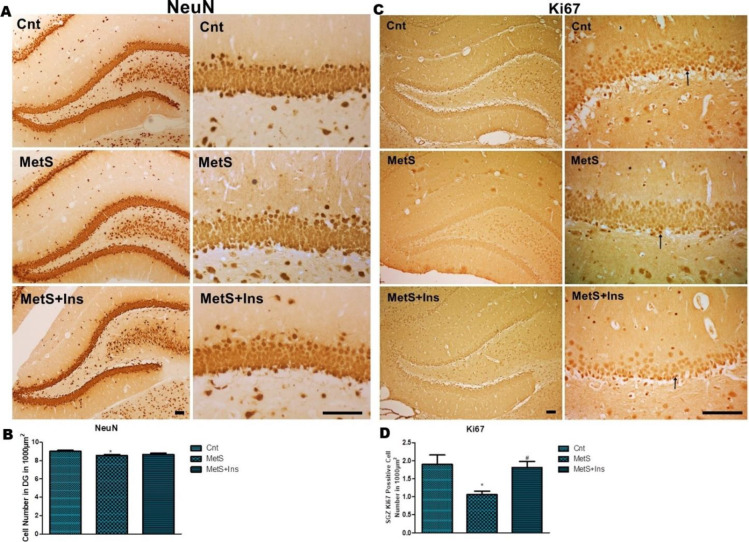
Immunohistochemical staining of the Ki67 and NeuN positive cells in the hippocampus. A) NeuN-positive cells in DG were stained dark brown. B) Positive cell number in 1000 µm^2^ in DG was shown in the graph. C) Ki67 positive cells in SGZ were stained dark brown and were shown with an arrow. D) Positive cell number in 1000 µm^2^ in SGZ was shown in the graph

Furthermore, a significant difference was found between the groups concerning the number of NeuN-positive cells in the DG area [F (2.27) = 4.075 *P*=0.028] (Figure 3A). The number of positive cells was reduced in the MetS group (*P*<0.05) (Figure 3B).

***Apoptosis was increased in MetS***

**Figure 4:**
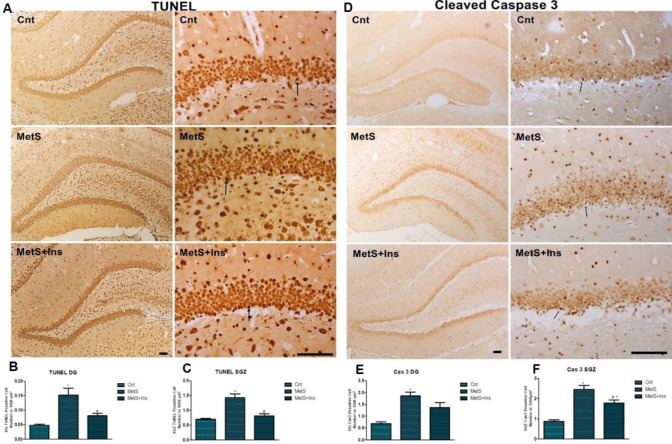
Immunohistochemical staining of the TUNEL and cleaved Caspase-3 positive cells in the hippocampus. A) TUNEL-positive cells in SGZ and DG were stained dark brown and were shown with an arrow. B) Positive cell number in 1000 µm^2^ in SGZ was shown in the graph. C) Positive cell number in 1000 µm^2^ in DG was shown in the graph. The graph was expressed as the means ± SD. *P<0.05 Control vs MetS-MetS+Ins, and #*P*<0.05 MetS+Ins vs MetS. D) Cleaved Caspase-3 positive cells in SGZ and DG were stained dark brown and shown with arrow. E) Positive cell number in 1000 µm^2^ in SGZ was shown in the graph. F) Positive cell number in 1000 µm^2^ in DG was shown in the graph

There were significant differences between the groups in terms of TUNEL positive cell numbers [SGZ; F(2.25)=26.784 *P*<0.0001, DG; F(2,28)=17,424 *P*<0.0001] (Figure 4A). A statistically significant difference was detected concerning the number of TUNEL-positive cells in the MetS group (SGZ; *P*<0.0001, DG; *P*<0.0001), while no difference was detected in the MetS+Ins group (SGZ; *P*=0.675, DG; *P*=0.156) compared with control. Additionally, a decrease was detected in the number of positive cells between MetS and MetS+Ins groups (SGZ; *P*<0.0001, DG; *P*<0.001) (Figure 4B-C).

There were also statistically significant differences among the groups in terms of cleaved caspase-3 immune-positive cells in SGZ and DG areas [SGZ; F (2.26) = 33.991 *P*<0.0001, DG; F (2.28) = 11.127 *P*<0.0001] (Figure 4D). A significant increase was detected in the number of positive cells in the MetS group (both SGZ and DG *P*<0.0001), while the number of positive cells was increased in only the SGZ area in the MetS+Ins group (*P*<0.001) compared with the control group (Figure 4E-F).

***Insulin signaling was impaired in MetS***

**Figure 5:**
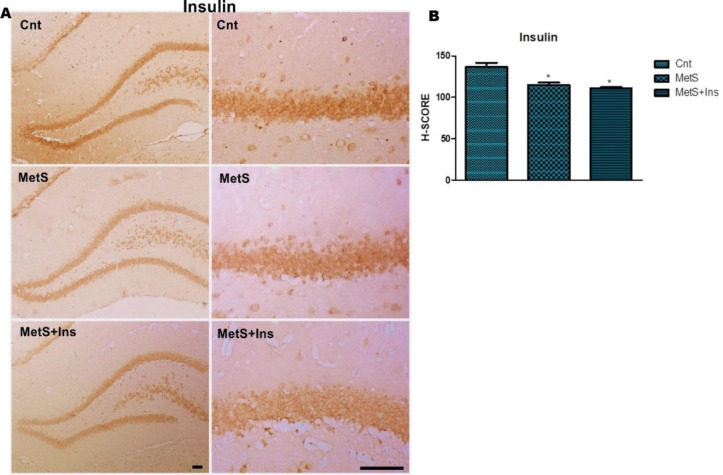
Immunohistochemical staining of the Insulin primary antibody in the hippocampus. A) Immunohistochemical labeling of insulin expression in the DG of the hippocampus. Representative photomicrographs were taken at magnifications of 10X and 40X, Bar: 100 µm. B) H-SCORE levels of immunostaining intensity of insulin in the DG region of the hippocampus

There were significant differences among the groups concerning insulin H-score values [F (2,30) = 19,353 *P*<0.0001] (Figure 5A). The mean of H-score in the control group was higher than in the other two groups (*P*<0.0001) (Figure 5B). 

**Figure 6:**
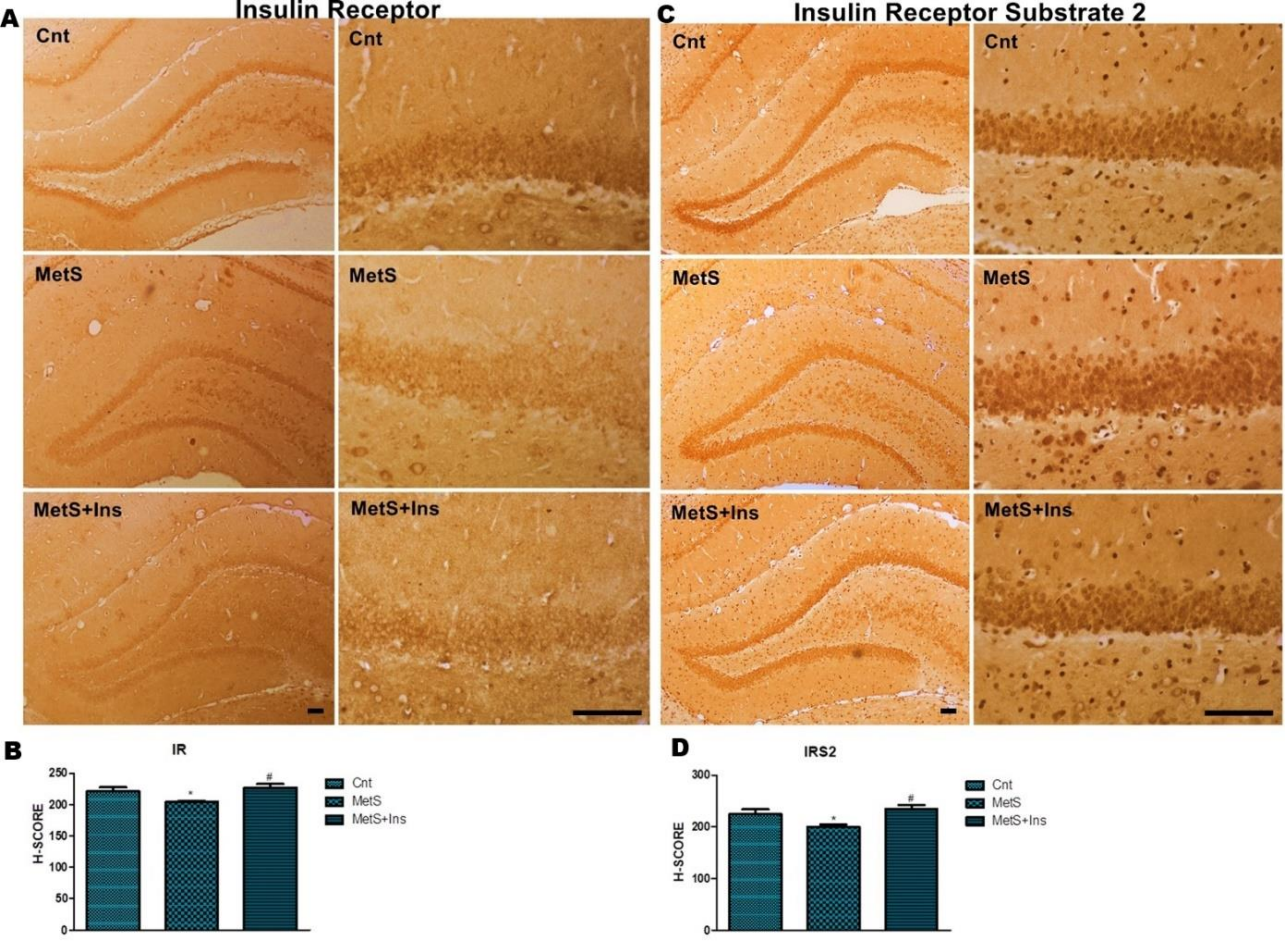
Immunohistochemical staining of the IR and IRS2 primary antibodies in the hippocampus. A) Immunohistochemical labeling of insulin receptor expression in the DG of the hippocampus. B) H-SCORE levels of immunostaining intensity of insulin receptor in the DG region of the hippocampus. The graph was expressed as the means ± SD. *P<0.05 Control vs MetS and #*P*<0.05 MetS+Ins vs MetS. C) Immunohistochemical labeling of IRS2 expression in the DG of the hippocampus. D) H-SCORE levels of immunostaining intensity of IRS2 in the DG region of the hippocampus. Representative photomicrographs were taken at magnifications of 10X and 40X, Bar: 100 µm

The H-score means of IR and IRS2 were different (*P*=0.006) (Figures 6A and 6C). Although a significant decrease was detected in terms of IR positivity in the MetS group (*P*<0.05), no statistically significant difference was detected in the MetS+Ins group (*P*=1.00) compared with the control (Figure 6B). Additionally, no statistically significant difference was detected between control-MetS and control-MetS+Ins groups concerning IRS2 positivity (*P*=0.065 and *P*=0.935, respectively). When the MetS and MetS+Ins groups were compared, it was observed that the mean H-score increased in the MetS+Ins group (*P*=0.006) (Figure 6D).

**Figure 7:**
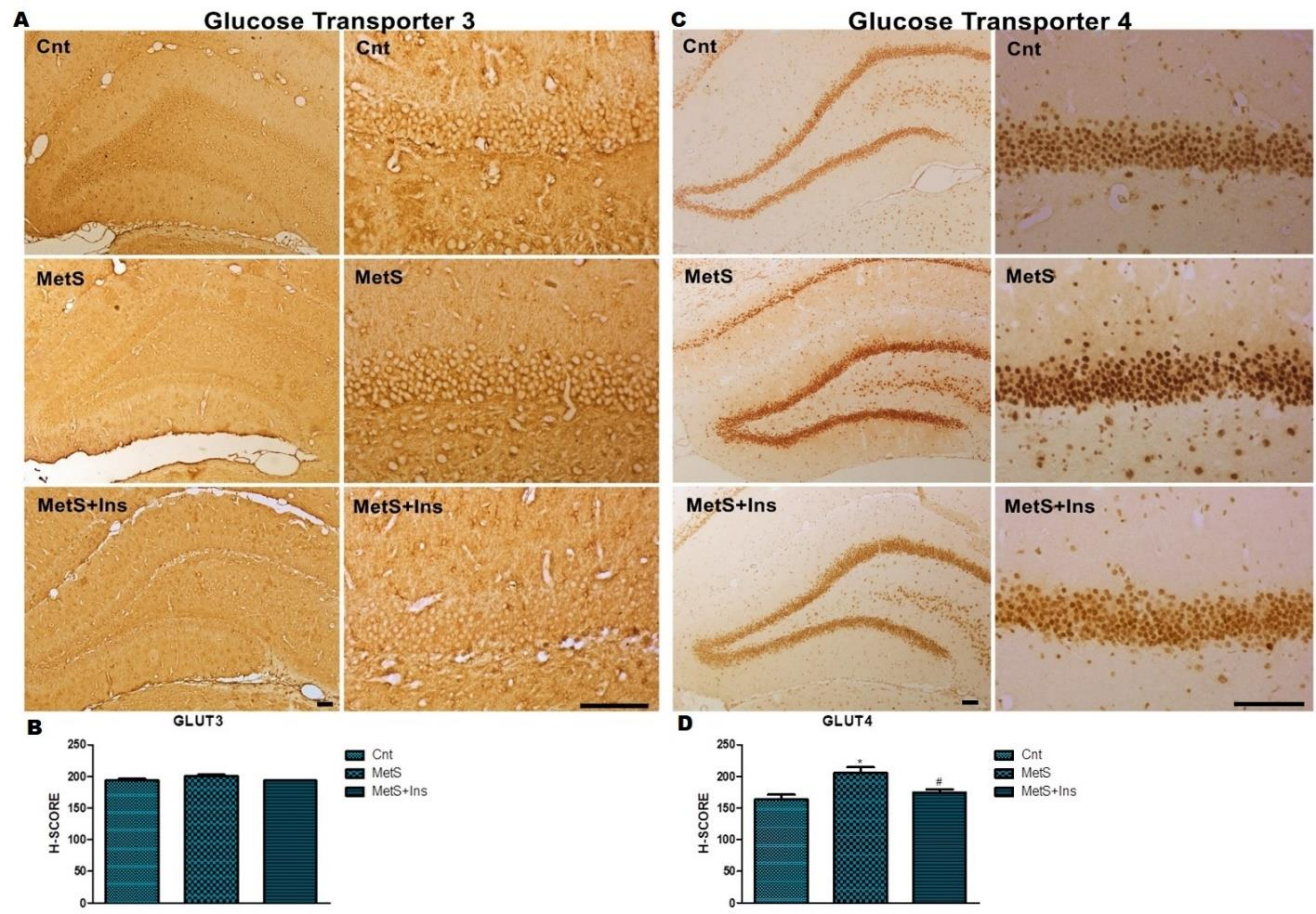
Immunohistochemical staining of the Glut3 and Glut4 primary antibodies in the hippocampus. A) Immunohistochemical labeling of Glut3 expression in the DG of the hippocampus. B) H-SCORE levels of immunostaining intensity of glucose transporter3 in the DG region of the hippocampus. The graph was expressed as the means ± SD. C) Immunohistochemical labeling of Glut4 expression in the DG of the hippocampus. D) H-SCORE levels of immunostaining intensity of glucose transporter4 in the DG region of the hippocampus. Representative photomicrographs were taken at magnifications of 10X and 40X, Bar: 100 µm

Although no statistical difference was detected concerning the mean of GLUT3 H-score values among groups (Figure 7A-B), a difference was detected in terms of the mean of GLUT4 H-score values between groups (*P*>0.05) (Figure 7C-D).

***Learning-Memory levels were decreased and Anxiety-like behaviors were increased in MetS***

EPM and Vanderwolf swimming tests were performed for evaluating learning-memory levels. It was observed that learning-memory levels were decreased in the MetS group when compared with the control group (Table 1) (*P*<0.05). EPM, OFT, and LDB tests were performed to evaluate the anxiety-like behaviors. It was detected that anxiety-like behaviors were increased in the MetS group when compared with the control group (Table 1).

Graphs were expressed as the means ± SD. *P<0.05 MetS vs Control and #*P*<0.05 MetS+Ins vs MetS

OGTT: Oral glucose tolerance test; MetS: Metabolic syndrome

Representative photomicrographs were taken at magnifications of 10X and 40X, Bar: 100 µm

Graphs were expressed as the means ± SD. *P<0.05 MetS vs Control and #*P*<0.05 MetS+Ins vs MetS

DCX: Doublecortin; D1: Differentiation-1; SGZ: Subgranular zone; MetS: Metabolic syndrome

Representative photomicrographs were taken at magnifications of 10X and 40X, Bar: 100 µm. Graphs were expressed as the means ± SD. *P<0.05 MetS vs Control and #*P*<0.05 MetS+Ins vs MetS

DG: Dentate gyrus; SGZ: Subgranular zone; MetS: Metabolic syndrome; NeuN: Neuronal nuclear protein

Representative photomicrographs were taken at magnifications of 10X and 40X, Bar: 100 µm. The graph was expressed as the means ± SD. *P<0.05 Control vs MetS-MetS+Ins, and #*P*<0.05 MetS+Ins vs MetS

TUNEL: Terminal deoxynucleotidyl transferase dUTP nick end labeling; DG: Dentate gyrus; SGZ: Subgranular zone; MetS: Metabolic syndrome

The graph was expressed as the means ± SD. *P<0.05 Control vs MetS-MetS+Ins

DG: Dentate gyrus; MetS: Metabolic syndrome

The graph was expressed as the means ± SD. *P<0.05 Control vs MetS and #*P*<0.05 MetS+Ins vs MetS

IR: Insulin receptor; IRS2: Insulin receptor substrate2; DG: Dentate gyrus; MetS: Metabolic syndrome

The graph was expressed as the means ± SD. *P<0.05 Control vs MetS and #*P*<0.05 MetS+Ins vs MetS

Glut3: Glucose transporter3; Glut4: Glucose transporter4; DG: Dentate gyrus; MetS: Metabolic syndrome

**Table 1:** Learning, memory and anxiety-like behavior test results in rats

**Behavior tests, reason for investigation**	**Behavior parameters**	**Control**	**Metabolic syndrome** **(MetS)**	**Metabolic syndrome + insulin (MetS+Ins)**
1. OFT ( horizontal locomotor activity)	Total distance traveled	828±382,76 **#**	344±256,26	746,0±393,7 *
2. OFT (vertical locomotor activity)	Total rearing number	10,8±5,84 **#^**	4,8±3,52	5,3±2,4
3. OFT (exploratory behavior)	Unsupported rearing number	4,7±2,21 **#^**	1,2±1,47	2,2±1,13
4. OFT (ALB)	Central zone time	16,7±12,05 **#^**	5,70±4,16	7,00±4,2
5. OFT (ALB)	Central zone entrance	1,2±1,61 **#^**	-	0,1±0,31
6. OFT (ALB)	Freezing time	35,5±11,65 ** #**	64±8,43	39±8,43 *
7. OFT (ALB)	Total grooming time	25,4±4,32	30±4,11	28±4,42
8. OFT (ALB)	Unsequential grooming time	7,2±2,34 **#**	14,6±1,83	9,2±1,68 *
9. EPM (ALB)	Open arm time	56,6±1,81 **#^**	14,8±8,67	40,9±19,09 *
10. EPM (ALB)	Open arm entrance	4±0,70 **#**	1,22±0,44	3,55±0,88 *
11. EPM (exploratory behavior)	Head dipping behavior	8±1,63 **#^**	2,2±1,03	4,5±0,97 *
12. EPM (ALB)	Stretch-attend posture	2,5±0,84 **#**	5,6±1,35	3,2±0,63 *
13. LDB (ALB)	Light zone time	35,2±39,91 **#**	11,0±5,16	31,7±13,74 *
14. EPM (learning and memory)	Transfer latency time	11,0±3,33 **#^**	63,7±15,03	32,1±6,15 *
15. Vander Wolf Swim Test (learning and memory)	Retention test swimming time	29,02±5,28 **#**	65,72±17,5	37,6±4,5 *
16. Vander Wolf Swim Test (learning and memory)	Acquisition test error number	4,1±0,87 **#^**	7,3±1,49	6±1,15
17. Vander Wolf Swim Test (learning and memory)	Retention test error number	1,2±0,42 **#^**	6,8±1,47	3±0,66 *

#: Control vs MetS group statistical significance, #^: Control vs both MetS and MetS+Ins groups statistically, *: MetS+Ins vs MetS group statistical significance. Significance value (*P*<0.05)

OFT: Open field test; EPM: Elevated plus maze; LDB: Light-dark box; ALB: Anxiety-like behavior

## Discussion

The obtained data reported an important decline in the levels of hippocampal neurogenesis in SGZ in MetS. This decline was demonstrated by IHC staining with DCX, NeuroD1, Ki67, and NeuN positivity. DCX is used to show the decreasing of neurogenesis in rats with type II DM (18), and Ki67 is used to prove the adult neurogenesis (19). On the other hand, it has been explained that NeuroD1 is necessary for maturation and survival of newly formed neurons in adult neurogenesis (20). A study claimed that NeuroD1 gene expression decreased in the hippocampus of rats fed with standard chow and 10% sucrose diet (21). In our study, we observed a decrease in the number of DCX, NeuroD1, and Ki67 positive cells in SGZ in MetS. 

To investigate, whether the reason for neurogenesis decline is related to apoptosis, Caspase-3 and TUNEL levels in SGZ were analyzed by IHC staining. The obtained data showed that the number of apoptotic neurons increased in SGZ in the MetS group, while the number of apoptotic neurons in SGZ in the MetS+Ins group decreased. In current literature, Ho *et al*. (22) pointed out that the levels of apoptotic markers increased in the hippocampus of diabetic rats. Another study reported that MetS increases the risk of type 2 DM by 5-fold (23). 

NeuN is only expressed in mature neurons and it has been noted that neurogenesis is reduced through deletion of this gene. The dysfunction of NeuN can be seen in epilepsy, autism spectrum disorder, neurodevelopmental delay, and cognitive disorders (24). To detect the reason for this decrease, we analyzed the cleaved Caspase-3 and TUNEL positive neuron numbers in DG and found that the level of apoptosis was increased in DG in the MetS group. Even though the number of NeuN-expressing cells was increased in the MetS+Ins group, this increase was not statistically significant when compared with the MetS group.

Hyperinsulinemia, which occurs with MetS, contributes to deterioration of glucose tolerance by causing an increase in insulin resistance (25). The aim of insulin administration to the experimental group is to examine its effects on neurogenesis by reducing the blood glucose level closer to euglycemic values. After insulin administration, the decrease in body weight, blood insulin, and OGTT levels in the MetS+Ins group was an indication of euglycemic values. These data were compatible with the current literature (14). The neurogenesis markers were high in the MetS+Ins group; however, this increase was not significant in our study. A significant decrease in the number of apoptotic cells was observed in SGZ where neuronal stem cells were located. Similarly, a statistically insignificant increase in the number of mature cells expressing NeuN was also observed.

In the central nervous system, insulin binds and phosphorylates IR, followed by phosphorylation of IRS. Phosphorylation of IRS activates the PI3K and MAPK/ERK pathways (26). It has been shown that PI3K signaling pathway is important for the metabolic effects of insulin and this pathway is generally affected in patients with MetS and DM (27). In type 2 DM patients, it has been shown that IR and IRS levels decrease with the formation of insulin resistance in the hippocampal region (28). Reduced insulin level leads to a decrease in the activation of insulin signaling. Then, the central control of peripheral metabolism is adversely affected and the rate of insulin resistance increases (29). In our study, the reduction in insulin, IR, and IRS2 expression levels was pointed out in especially DG of MetS groups. Insulin has been shown to regulate the proliferation and differentiation processes of hippocampal stem cells and plays a protective role against apoptosis and oxidative stress (30). The reason for the decrease in neurogenesis in MetS may be disruption of the insulin signaling pathway due to insulin resistance. Our findings reported that expression levels of neurogenesis proteins, IR, and IRS2 were elevated in the MetS+Ins group.

Glucose is the main energy source for the brain. The uptake, transport, and use of glucose into neurons are affected by insulin, but is not insulin-dependent (31); although most glucose uptake in neurons occurs via GLUT3, which is co-expressed with insulin-regulated GLUT4 (32). In our study, although there was no significant difference, GLUT3 expression was increased. A study reported that GLUT4 expression in the cell membrane was decreased by reduced insulin signaling and impaired glucose metabolism in rat brains (33). In general, brain insulin signaling and regulation of GLUT4 may differ in peripheral tissues (34). In our study, the GLUT4 expression level was increased in the hippocampus of rats with MetS, despite insulin resistance. This can be explained in two ways. First, the regulation of GLUT4 in the hippocampus is indeed different from the peripheral tissues. Second, there is increased cytoplasmic expression and decreased membrane localization of GLUT4. At that point, our study has a limitation in that we should have determined whether the increased GLUT4 expression originated from the cytoplasm or localization to the membrane.

Nowadays, due to the rapid change in eating habits, most related studies have been examining the effects of high-fat and sucrose diets on behavior. Whether high sucrose intake changes anxiety-like behaviors, learning and memory is controversial. It has been shown that anxiety-like behavior is not related to the consumption of high sucrose diet, whereas motor learning impairment is related (35). Another study showed that 25% sucrose intake increased anxiety-like behaviors (36). The results in our study cover the data in those two studies. We detected that anxiety-like behaviors increased and learning-memory skills decreased in the MetS group. It is known that the hippocampus has a role in learning, memory, and spatial orientation. In our study, we showed that neurogenesis-related protein levels were decreased, apoptosis was increased, and the mean neuron numbers were decreased in the hippocampus. If neurogenesis is reduced in the hippocampus, learning and memory decline is an inevitable outcome. Additionally, deletion of IRS2 in male mice reveals a negative effect on hippocampus-related emotional responses and spatial memory (11). It is reported that IR and IGF1R are important for mood and cognition in the hippocampus and central amygdala (10). Parallel with the current literature, we detected that IR and IRS2 expression were decreased in the MetS group. These results support the increase in anxiety-like behaviors in MetS rats. Elevations in the levels of IR, IRS2, and neurogenesis markers after insulin administration support a decrease in anxiety-like behaviors and an increase in learning and memory in rats.

## Conclusion

This study showed a significant decrease in neurogenesis and an increase in apoptosis. Also, the levels of some proteins involving insulin signaling pathways were changed in the hippocampus regions of rats with MetS. As a result of these changes, anxiety-like behavior was increased, while memory and learning rates were decreased. In light of these data, we think that the insulin signaling pathway may have an effect on the decreased neurogenesis in the MetS group. For this reason, evaluation of the MAPK pathway and investigation of the effect of ER stress on this pathway will be among the targets of our future studies. 

## Authors’ Contributions

BP and DB Conceived the presented idea. KS, CB, and CH Developed the theory. All authors performed the experiments. BP and KS Verified the analytical methods. CH and BP Performed behavioral experiments. All authors evaluated the laboratory data and contributed to writing the paper.

## Conflicts of Interest

The authors have no conflicts of interest
